# Ensuring Safe Newborn Delivery Through Standards: A Scoping Review of Technologies Aligned with Healthcare Accreditation and Regulatory Frameworks

**DOI:** 10.3390/healthcare14030377

**Published:** 2026-02-02

**Authors:** Abdallah Alsuhaimi, Khalid Saad Alkhurayji

**Affiliations:** 1 Executive Department of Standards, Saudi Central Board for Accreditation of Healthcare Institutions, Riyadh 12264, Saudi Arabia; aalsuhaimi@cbahi.gov.sa; 2 Research, Statistics, and Information Department, Saudi Central Board for Accreditation of Healthcare Institutions, Riyadh 12264, Saudi Arabia

**Keywords:** accreditation, infant, newborn, public health, global health, infant health

## Abstract

**Background/Objectives:** Safe delivery and correct identification of newborns are critical aspects of healthcare systems globally. The accreditation of healthcare and standards regulation significantly promotes the adoption of modern technologies to address risks related to infant abduction and misidentification. The effectiveness and extent of these mandates vary across settings and countries. Therefore, this study aims to map and explore modern technologies used for safe newborn delivery and correct identification aligned with healthcare accreditation and regulatory frameworks. **Methods:** This review adheres to the Preferred Reporting Items for Systematic Review and Meta-Analysis extension for scoping reviews (PRISMA-ScR) guidelines. The Problem, Intervention, Comparison, and Outcome (PICO) framework was employed to facilitate the development of the research question. This study examined studies reporting technologies such as radio frequency identification (RFID), biometric identification, and real-time monitoring across healthcare settings for infant protection through the Normalization Process Theory (NPT). Among three databases and search engines (PubMed, Google Scholar, and Web of Science). The risk of bias for each study was assessed using the AACODS Checklist, SQUIRE 2.0 Checklist, TIDieR Checklist, and JBI tools. **Results:** Out of 8753 records, only 27 reports were eligible to be included in this review. The most frequently reported technologies were RFID systems (11 studies, 37.9%) and biometric systems such as footprint and facial recognition (6 studies, 20.7%). Despite strong technological potential, many healthcare institutions struggled with the adoption of infant protection technologies. Accreditation systems among the high-resource settings actively mandate advanced technologies and support the integration of staff training and simulation drills. Comparably, middle- and low-income regions usually face challenges related to regulatory enforcement, infrastructure, staff readiness, and limited adoption of modern technologies. **Conclusions:** Accreditation and standards development are critical catalysts for the adoption of modern infant protection technology. Standards must be comprehensible, adaptable, and supported by investment in human resources and infrastructure. Future regulation must focus on strengthening enforcement, continuous quality improvement, and capacity building to achieve sustainable protection across the world.

## 1. Introduction

Safe delivery of the infant and accurate identification immediately post-birth is considered an imperative priority recognized and upheld by the healthcare systems across the world, which reflects a universal commitment to protect the most vulnerable members of society [1,2]. The alarming challenge of infant misidentification and infant abduction presented significant threats to the overall safety and well-being, which can lead to a myriad of severe physical, psychological, and social impacts extended to the families, in addition to the healthcare providers who were entrusted with the protection and care of these infants [3,4].

In responding to these pressing critical challenges, healthcare accreditation systems and regulatory bodies increasingly emphasized the urgency of adopting and integrating modern technologies, including but not limited to RFID, advanced real-time monitoring mechanisms, and biometric identification systems, to improve and maintain the security measures surrounding infant care [5,6,7]. The innovative technologies possess the potential for significant enhancement of the security of infants through facilitating precise tracking capabilities, minimizing the likelihood of human error, and enabling a swift and efficient response to any threats that may occur during the critical early stages of life [8,9]. Nevertheless, various accreditation entities mandate and enforce the implementation of certain advanced technologies, exhibiting variability across different geographical regions and diverse healthcare institution settings, which could lead to inconsistencies in the safe development protocol of infants [10,11]. Furthermore, the ranges of challenges, such as existing infrastructure, organizational readiness, and adequacy of healthcare providers’ training to adopt new technologies, can serve as critical barriers impacting the effective adoption and long-term sustainability of these essential safety precautions [12,13].

By gaining a comprehensive understanding of these dynamics, paramount importance is reflected in informing the development of up-to-date policies, enhancing the healthcare standards accreditation, and ultimately ensuring that the risk is minimized in terms of abduction, mismatching, and improving the overall safety and security of infants across different healthcare contexts. Therefore, this study aims to provide a mapping and systems-level overview, rather than comparative effectiveness estimates. Subsequently, answer the research question: To what extent are modern technologies used for safe newborn delivery and correct identification aligned with healthcare accreditation and regulatory frameworks?

## 2. Materials and Methods

This systematic review adheres to Preferred Reporting Items for Systematic Review and Meta-Analysis extension for scoping reviews (PRISMA-ScR) guidelines (See Supplementary File S1) [14]. The problem, Intervention, Comparison, and Outcome (PICO) framework was employed to facilitate the development of the research question [15], as illustrated in Table 1.

### 2.1. Eligibility Criteria

All observational studies encompassing all varieties, including cohort studies, case–control studies, or cross-sectional studies that examined the application of contemporary technologies for enhancing infant safety (e.g., RFID, barcode, biometric identification, smart bracelets, infant security systems), either with a formal requirement, standard, directive, or safety expectation or recommendations to the healthcare facilities to implement newborn technologies or mandated or promoted by accreditation standards, in addition to facilities devoid of such mandates or employing manual/traditional identification practices, were included in this review. Nonetheless, non-human primate observational research and descriptive media-based perpetrator analyses were included to improve the coverage of the gray literature. We systematically searched regulations, policies, institutional reports, guidelines, and/or professional association publications relevant to newborn safety technologies. These sources were systematically extracted and mapped alongside peer-reviewed studies to provide a comprehensive overview. The inclusion criteria were restricted to studies involving technologies or methods for newborns within healthcare facilities, specifically those related to delivery, maternity, or neonatal units.

In this scoping review, regulatory requirements are defined as legally mandated obligations issued by governmental or legal authorities, while accreditation standards are formally assessed criteria established by recognized accrediting bodies, and recommendations are best-practice statements or non-binding guidance issued by professionals and/or organizations. The primary outcome of this review was the enhancement of infant safety, defined as a composite concept encompassing specific, observable indicators characterized by a reduction in mismatches, diminished abduction risk, improved delivery processes, and accurate mother–baby pairing, as reported in studies published in English or Arabic languages. Nevertheless, our exclusion criteria will not encompass reports that are not pertinent to infant safety, studies that concentrate on children younger than infants, or research conducted beyond the confines of hospitals and healthcare institutions.

### 2.2. Search Strategy

The literature searches were conducted utilizing PubMed, Web of Science, and Google Scholar. The temporal scope of the searches extended from the inception of these databases, starting from 1 January 2000, up to 20 May 2024. The elements of the search strategy encompassed in the search string included both MeSH terms and other relevant subject terminology, along with synonyms and search filters, which are detailed in the Supplementary File S2. The construction of the search strings was undertaken by a qualified librarian. In order to enhance our prospects of identifying all pertinent studies, in addition to querying databases, we also performed a manual examination of the reference lists from the studies that were included.

### 2.3. Study Selection and Screening

**Figure 1:**
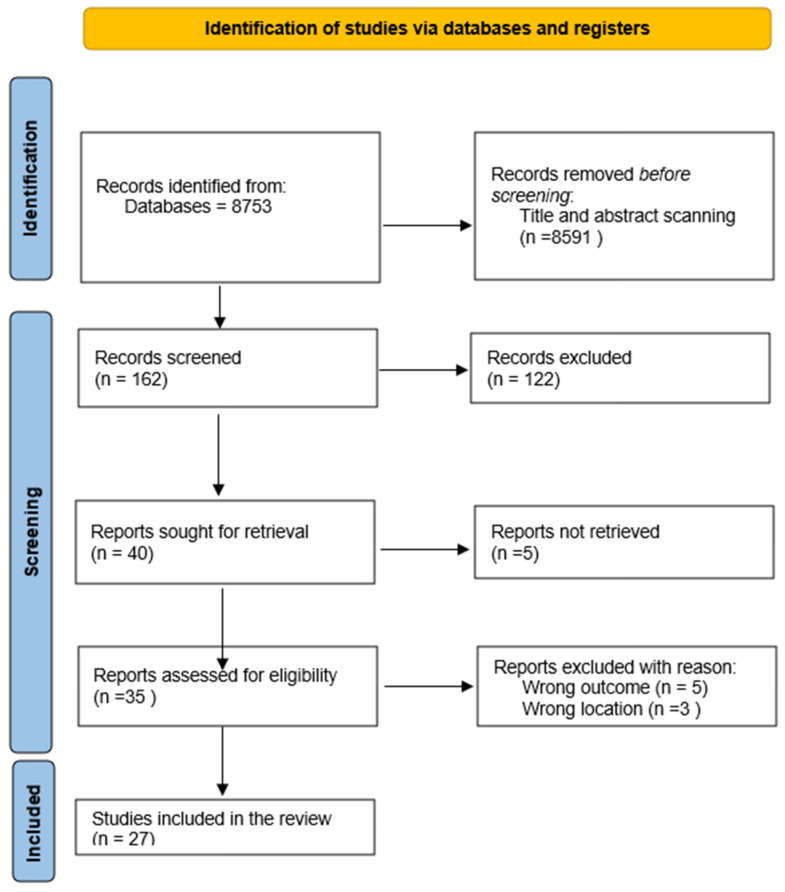
Flowchart of the study selection process.

The authors (K.S.A., A.A.) independently performed the screening of titles and abstracts. Subsequent to the screening of titles and abstracts, full texts of the remaining articles were procured by K.S.A. and A.A. The authors (K.S.A., A.A.) conducted an evaluation of the full texts in accordance with the established inclusion criteria. A citation search was undertaken by K.S.A. Any discrepancies that arose were addressed through censuses. The procedure for screening and inclusion is illustrated in Figure 1.

### 2.4. Data Extraction

We used a data extraction form for study characteristics and outcome data, which was piloted on five studies in the review. Study authors (K.S.A., A.A.) extracted the following data from included studies:

Types of studies: observational studies (all types), cohort studies, case–control studies, and cross-sectional studies.

Year, country, study design, setting, population characteristics, technology type, outcome measures, results summary, and limitations of the study.

### 2.5. Assessment of the Risk of Bias (RoB)

Given the heterogeneous nature of the included reports, multiple appraisal tools were used to assess reporting quality and methodological rigor across different evidence types. Tools were matched to report types based on relevance and guidance provided by each framework, ensuring appropriate and accurate assessment criteria were applied for each evidence source. Additionally, risk-of-bias findings were not used to exclude studies or quantitatively weight results, consistent with scoping review methodology. Instead, they were descriptively summarized and informed the interpretation of evidence, highlighting gaps in reporting quality and potential limitations. Two review authors (K.S.A. and A.A.) assessed the risk of bias for each study using the AACODS Checklist, SQUIRE 2.0 Checklist, TIDieR Checklist, and JBI (See Supplementary File S3) [16,17,18,19].

### 2.6. Dealing with Missing Data

In instances where data are absent, the authors of the study are engaged in order to obtain the missing information.

### 2.7. Synthesis of the Results

The Normalization Process Theory (NPT) was used as an analytical lens to interpret how accreditation-aligned or independently adopted technologies were implemented and embedded in routine practice, given the focus on the mechanisms through which such technologies are comprehended, embraced, and assimilated into standard clinical practice, particularly under the auspices of accreditation or regulatory frameworks. Thematic analysis, following Braun and Clarke’s six-step approach, was applied deductively using NPT, enabling the identification and structuring of themes corresponding to the key findings [20,21]. The data presented in this review include tabular, graphical, and narrative formats. In this synthesis, reports are categorized according to the type of technology implemented, setting, population, and presence or absence of accreditation mandates. Evidence is synthesized narratively rather than quantitatively, given the heterogeneous contexts.

## 3. Results

Figure 1 illustrates the meticulous delineation of the comprehensive systemic process by which studies were selected for inclusion in this review, commencing with 8753 records sourced from a diverse range of search engines and databases. The following stage included the removal of duplicated records as well as titles and abstracts, resulting in a reduction of 8591 records. Subsequently, 162 records were screened, which, ultimately, after post-screening, showed that only 122 records were eligible. The remaining 40 reports were pursued for retrieval from their respective sources. However, five of these reports could not be retrieved for further full report screening. The following stage included 35 reports, which were assessed for eligibility, resulting in the removal of 5 reports based on incorrect outcomes (technologies and recommendations reported for non-safe delivery for newborns) and 3 for the wrong location (technologies for non-healthcare settings). Finally, 27 studies were included in this review.

Table 2 shows that across the reviewed reports, RFID technologies emerged as the most commonly used solution in reports for infant identification and security measures in healthcare settings. Nonetheless, the majority of the studies from diverse locations in the world, such as India, Saudi Arabia, the US, Pakistan, and Taiwan, employed RFID systems to prevent mismatching, track infants, and detect unauthorized movement in hospital settings. Studies collectively reported that RFID improves monitoring accuracy and supportive initiatives of hospital safety despite the limitations in technical aspects and user acceptance that remain challenging.

On the other hand, biometric identification technologies such as facial recognition and footprint imaging were considered the second most frequently used and explored, particularly in India. Demonstrating accuracy with biometric systems suggests the potential for demonstrating accuracy. However, these technologies face practical barriers, such as data acquisition challenges, limiting their current widespread application, and infant non-cooperation.

In terms of geographical distribution, most studies have been conducted in the US and India, showing a strong focus in these countries on infant security and technologies. Reports conducted in the US often emphasize policies, accreditation standards, and simulation staff training or drills. While in Indian studies, which tend to focus, for instance, on the development and technical validation of RFID and biometrics systems. Other regions, such as Saudi Arabia, Pakistan, and Taiwan, provide valuable regional perspectives in terms of human factors but are fewer in number.

Regarding the approach of reports included in this review, which include a wide variety of aspects ranging from the development and validation of technologies to cross-sectional and policy analysis. The majority of technical approaches involve simulation or controlled environments rather than an ongoing need for field validation, while human and organizational factors, such as healthcare provider training, accreditation adherence, and simulation drills, are consistently highlighted as a critical complement in protection measures for infant safety.

Table 3 illustrates the reliance on RFID and biometric system technologies as the cornerstone of modern infant protection and identification systems. Nonetheless, emerging AI-based computer vision solutions and cybersecurity protocols complement those technologies. Given the varied use of simulation, physical security measurement, and policy analysis approaches, a holistic combination of technologies, human factors, and regulatory frameworks increases safety and minimizes risk. Several reports depicted newborn security technologies without explicitly referencing regulatory pathways, device certification, or compliance issues, which emphasizes a gap in reporting rather than an absence of governance. Collectively, these findings underline the need for enhancement of reporting in terms of certification and regulatory status in future studies evaluating safe newborn security technologies. Nonetheless, certain reports only assessed feasibility or implementation potential, which are reported as such, without inferring clinical outcomes. This highlights that the existing gaps in evidence and reporting, specifically regarding real-world effectiveness, and points out the need for future studies to evaluate technologies under routine clinical practice.

### 3.1. Coherence

Several studies highlighted that gaps in this theme, particularly with the end users, lacked the training necessary and the context needed to grasp the system’s value. For instance, according to Al Osaimi, Al Kadi, and Saddik [28].

“65.8% of nurses perceived RFID effective for tracking”, “only about 50% accepted the system”, and the reason was “inadequate training and computer skills.”

This reflects a disconnect between personal readiness and operational usage. Furthermore, according to Samayawardena [30], laws exist to protect infants from abduction:

“legal loopholes, inadequate hospital regulations, poor knowledge among relevant authorities.”

This domain of coherence remains a critical issue in contexts where legal, clinical, and technical systems fail to align with the shared understanding of risk and protection.

### 3.2. Cognitive Participation

The engagement of stakeholders is considered essential for the sustainability of security intervention in driving best practice, and this can be observed in the studies that promoted collaboration and simulation-based engagement. For instance, Shogan [45] asserts that “regular drills and education enhanced staff preparedness.” In contrast, according to Batool and Fatima [25], Pakistan reported alarmingly low levels of institutional engagement, where only 2.85% of hospitals are highly prepared and more than 85% lack basic readiness. This domain shows cognitive disengagement at the organizational level, which illustrates that technology alone is not enough, requiring normalization of the process and ongoing participation.

### 3.3. Collective Action

Among the highly integrated systems, safety protocols and technology were applied. According to Shilaskar and Lothe [22], who reported that the computer vision and deep learning in maternity hospitals achieved “85% classification accuracy”, likewise, Bittle and Scalise [27] study shows that implementing the green pass system ensured “no infants were discharged without completing discharge process.”, which clearly resulted in a safety standard of the discharge procedure. However, systems may face failure in practice when exposed to collective action. According to Goodwin [46], “both infant sensor bracelet and security door system failed”, underscoring how vulnerable health facilities’ systems remained when procedures were poorly integrated or inconsistently followed. Effective action, therefore, depending on synchronized implementation, is required across healthcare providers, infrastructure, and organizational workflow.

### 3.4. Reflexive Monitoring

The effectiveness of health systems includes incorporating feedback to adapt and improve over time. For instance, according to Webster and Stikes’ [26] report, which used a formal risk assessment study that identified 11 high-risk failures across 32 process steps, reflecting a proactive mitigation through Failure Mode and Effects Analysis (FMEA). Similarly, Hung, Chu [32] report that RFID is not the only prevention of identity error and abduction of infants. Asserted that supported compliance with the Baby-Friendly Hospital Initiative [23], which provides a dynamic approach through simulation to find communication flaws, ultimately improving that simulation training, *“revealed compliance issues”*, in addition to the need for personalized training. These reports collectively highlight how interventions improve from ongoing scrutiny and user feedback [31].

### 3.5. Reflexivity

The capacity of a health facility with adequate resources in terms of technical, financial, and human workforce can support intervention success. Despite the development of technologies’ potential, many healthcare systems struggled with infrastructure readiness. In RFID systems, which require encryption, network isolation, and stronger passwords, can improve surveillance and mitigate risk. For instance, Yadav and Pandey [29] reported that their RFID system reached 100% accuracy during testing. However, certain issues of security and scalability concerns were observed. Likewise, according to Kiruthiga and Birinda [24], who emphasized that while their prototype system showed significant potential, it required close monitoring to avoid technical failure. The findings collectively suggest that while innovation in technology is presented, its effective usage depends on whether the health system can provide and adapt in hospital settings and train the healthcare providers for such technology for sustainability.

Stakeholders’ point of view reflects certain interventions’ long-term value and whether they inspire future application or expansion in healthcare systems. The results are promising, given that the biometric footprint system showed it was much more accurate than traditional offline methods [33]. Furthermore, in facial recognition reports [34], the accuracy of face detection in newborn faces is indistinguishable, achieving 87.04% accuracy, which may open pathways for broader safety of infant applications. Nonetheless, according to Crémoux, Boyer, and Dhia [31], water immersion minimally affects RFID tag reliability, highlighting the practicality of this technology in real-world, significant applications. Despite the positive findings, many healthcare systems remain underutilized or stuck at pilot testing stages due to challenges in funding, policy, and a lack of required support.

The reviewed reports illustrate limited normalization into routine clinical practice, even though accreditation standards require the adoption of infant identification and safety technologies. Implementation failures were primarily caused by flaws in several NPT constructs rather than just technical shortcomings. Nonetheless, according to several reports, staff members’ limited comprehension of the clinical benefits of identification technologies beyond regulatory compliance results in their superficial or symbolic use at the coherence level. The lack of local champions and low frontline involvement in implementation processes often limited cognitive participation, lowering professional ownership. Furthermore, workflow disruption, increased documentation burden, inadequate training, and insufficient technical support, specifically in low-resource settings, were all frequently noted obstacles to collective action. Lastly, reflexive monitoring was inadequate throughout, given that reflexive monitoring was inadequate in the majority of reports, with few feedback mechanisms to show improvements in safety or a decrease in errors, which undermined sustained engagement. When taken as a whole, these results show that accreditation requirements by themselves are not enough to guarantee normalization. However, successful embedding requires consistent teamwork across all four NPT constructs.

## 4. Discussion

This review revealed that healthcare accreditation systems and regulations play a pivotal role in mandating the implementation of modern technologies to ensure the safe delivery, in addition to the correct identification of the infant. Across the review of the literature, there were clear reports that accreditation bodies, particularly in the US, actively require health facilities to adopt advanced security measures such as RFID tracking systems, biometric identification, and comprehensive protocols supported by policies and regulations.

The accreditation standards in healthcare usually drive hospitals to integrate technologies with rigorous workforce training programs and simulation drills, which enhance preparedness and reduce vulnerabilities [3,23]. However, in low- and middle-income settings, the regulatory requirement remained either poorly enforced or weak, resulting in wide disparities in technology adoption for infant security preparedness [25,28]. Furthermore, even for such accreditation mandates that exist, the successful implementation depends heavily on contextual factors such as infrastructure capacity, ongoing evaluation and monitoring, staff acceptance, and, reflecting the regulatory pressure, is insufficient alone without a supportive organization. While accreditation and standards in healthcare serve as an essential component for modern technology use in newborn safety, their impact varies substantially by location and organizational readiness, underscoring the need for more comprehensive, enforceable, and context-sensitive policies.

Collective reports highlighted that healthcare institutions worldwide are increasingly adopting advanced technologies and safety protocols to safeguard infants and prevent abduction or mismatch. A major theme that emerged from the review was the reliance on RFID-based systems and biometric technologies as an essential frontline tool for infant tracking and identification. Multiple reports from Saudi Arabia, the US, Taiwan, and India demonstrate that RFID systems can significantly improve monitoring, reduce mismatching, and minimize abduction risk [28,29,32]. However, these systems usually face challenges related to technology acceptance among healthcare providers, technical failure, and environmental factors like signal interference, which could compromise their effectiveness. The limitation of successful implementation also required workflow integration and employee training [28,40]. However, beyond RFID, biometric methods such as infant footprint imaging and face recognition algorithms provide promising potential, with reported results showing high accuracy and reliability rates [33,35]. These biomaterials provide a valuable layer of security prevention, especially in sensitive hospital environments such as NICUs. Nonetheless, simulation training and adherence to accreditation standards play vital roles, given that simulation drills and policy compliance significantly improve staff preparedness and protocol adherence, thereby reducing vulnerabilities such as unauthorized access and communication breakdowns [3,23]. These technologies must be paired with well-designed training initiatives and effective policies in the organization. In addition, the involvement of teams and continuous quality improvement projects further supports the role of safeguarding infants and supports the critical role of systemic factors in safeguarding infants [27].

Considering the gaps that remained, especially in low-resource settings, hospitals’ preparedness for infant protection is generally poor, reflecting a lack of security measures, infrastructure, and national policies [25]. Similarly, enforcement of policies and legal consideration documents in Sri Lanka emphasizes the need for stronger regulatory frameworks and a better awareness level among the healthcare providers and authorities [30]. These findings from the reports showed inequality in infant security provision and measurement and called for context-specific intervention.

Despite the value presented by RFID systems, technical vulnerabilities and the critical risk of system failure pose an ongoing challenge. Several reports showed that RFID systems and security alarms are usually susceptible to tampering, hacking, and false alarms when the system is not properly encrypted or monitored by adequate human oversight [40,46]. Moreover, reliance solely on technology.

In summary, the current review recommends a multifaceted approach combining technological innovations, in particular RFID and biometrics, rigorous training, simulation, accreditation standards, policy adherence, and robust legal frameworks for effective infant safety. While advanced AI tools and computer science are promising [22], the future work needs to prioritize validation, provide tailored solutions to local resources and policy environments, and address staff acceptance barriers.

This study has certain limitations. First, the variability in resource availability, healthcare settings, and regional regulations across the included studies may limit the generalizability of our findings. Additionally, certain reports relied on testing projects or early-stage prototypes, which may not reflect actual effectiveness. In addition, the lack of standardized outcome measurement in the reporting of the studies hindered the ability to conduct syntheses quantitatively or perform a meta-analysis.

To the best of the authors’ ability in terms of language, reports in Arabic and English were retrieved and included in this study. However, future studies need to consider inspecting additional languages to further expand the literature coverage. Despite this limitation, the English language captures most of the international evidence in terms of regulatory documents, accreditation standards, and peer-reviewed studies, while limiting the Arabic and English languages can ensure accurate data extraction and syntheses, given that translation from other languages could lead to errors or misinterpretation, given the complexity of regulatory and technical content.

While this scoping review makes an effort to provide a global perspective, the included reports mainly represent certain regions, with limited available evidence. Additionally, accreditation standards and regulatory frameworks vary in general across countries, and low-resource settings encounter different challenges in implementing newborn safety and identification technologies. As a result, interpreting the results should be performed with caution, and future direction should focus on capturing region-specific accreditation experiences and regulatory frameworks, particularly in underrepresented regions, to inform context-sensitive policy and implementation strategies. For future research, longitudinal studies need to be executed to assess the impact of accreditation mandates on infant safety outcomes and to investigate strategies to enhance staff engagement and training, as well as evaluate the cost-effectiveness of these technologies.

## 5. Conclusions

Healthcare accreditation bodies and regulatory frameworks play a crucial role in driving the adoption of technologies aimed at ensuring the safety of infants in hospital settings and accurate identification, minimizing the risk of abduction and mismatch. While these frameworks effectively advance security measures, there is still a significant gap in the implementation, where training, regulatory enforcement, and infrastructure are often insufficient. Successful integration of technologies depends on regulatory requirements in addition to staff engagement, ongoing evaluation, and alignment of legal and healthcare organization context.

To maximize the potential success of accreditation standards in the protection of infants, future efforts must focus on creating comprehensive, adaptable policies that address contextual issues and challenges in addition to protecting sustainability, which will advance and reduce the risk of accreditation and mismatching worldwide.

## Figures and Tables

**Table 1:** Items description of the PICO framework.

PICO Component	Description
P (problem)	Safe delivery and correct identification of newborns in healthcare facilities (delivery, maternity, or neonatal units)
I (Intervention)	Application of modern technologies for infant safety (e.g., RFID, barcode, biometric ID, smart bracelets, infant security systems), mandated or promoted by accreditation standards
C (Comparison)	When applicable, facilities with no such mandate, or using manual/traditional ID practices
O (Outcome)	Improved infant safety—fewer mismatches, reduced abduction risk, safer delivery processes, and correct mother–baby pairing

**Table 2:** Key characteristics of included studies.

Reference	Country	Approach	Technology	Setting and Population	Outcome Measures	Main Findings	Mandates	**Limitations**
[22]	India	Development and validation	Computer vision (HOG, SVM), deep learning (LSTM), and machine learning algorithms for activity recognition and crowd detection	Maternity hospitals; infants, mothers, staff	Classification accuracy for detecting “with baby” vs. “without baby” (~85%), action recognition accuracy (~78%), and success in identifying suspicious activities	The system effectively detects infants and suspicious activities in real-time, demonstrating promising performance in simulated hospital environments	Not stated	Dataset limitations: sensitive to lighting/activity changes
[3]	United States	Literature review + simulation drills	RFID systems, surveillance cameras, biometric infant ID tools, and physical security measures	Hospital facilities; infants, staff, impersonators	Simulation drill outcomes, incident reports, staff preparedness, risk vulnerabilities, protocol adherence (e.g., access control, response times)	Accreditation and adherence to security policies improve staff response and reduce vulnerabilities; key vulnerabilities include tailgating, impersonation, communication gaps, and staff challenge weaknesses	The Joint Commission requires healthcare facilities to implement effective staff training and critical incident response procedures to ensure physical security, protect patients from external threats, and prevent sentinel events	US-centric; reliance on reported/simulated data
[23]	United States, Israel	Modified Delphi and simulation	Simulation technology for training handovers	Pediatric and emergency settings; healthcare workers	Simulation drill outcomes, incident reports, staff preparedness, risk vulnerabilities, protocol adherence (e.g., access control, response times	Identified five core competencies for medical command and control; simulation training improved handovers; personalized training needed to address communication gaps; simulation reveals compliance issues	Not stated	Scope limited; training not evenly implemented
[24]	–	Technical system design	RFID tags, sensors, microcontrollers	Neonatal hospital settings	Effectiveness of the RFID system in preventing infant abduction; alerting staff to unauthorized movement	The prototype shows significant potential in improving neonate security by detecting unauthorized access and triggering alerts	Precluding a regulation, Pink is thus a pinnacle priority that requires preceding planning, cautious format of bodily walls, and the perpetration of the proper applied sciences	Needs monitoring; tech failure risks; compliance needed
[25]	Pakistan	Cross-sectional hospital survey	Focus on hospital preparedness/security measures	Postnatal wards; mothers	Hospital preparedness levels are categorized (highly prepared, less prepared, unprepared); factors influencing security measures	Only 2.85% of hospitals are highly prepared; 85.72% unprepared; 73.1% mothers recognized the need for enhanced security	modified questionnaire. This tool is recognized for its guidelines on preventing and responding to infant abductions, and guidelines from the National Center for Missing and Exploited Children (NCMEC)	No tech studied; lacks national policy; limited scope
[26]	United States	FMEA risk assessment	ID bracelets; sensor range	Women’s and Infants hospital unit	Risk Priority Numbers (RPN) of failure modes in infant identification	Identified 32 process steps and 28 failure modes, with 11 high-risk failures; FMEA implementation improved patient safety and security awareness	As one of only 4 facilities in Kentucky to be designated a Baby-Friendly birthing facility, the CWI strives to improve performance and prevent harm to patients.	Some safety practices were excluded due to a low-risk rating, possibly missing other concerns
[27]	United States	Interdisciplinary quality improvement	Green pass system linked to footprint identification for discharge verification	Newborns in NICU and their parents	Completion of the discharge process and prevention of infant abduction	Since green pass implementation, no infants have been discharged without completing the discharge process; adherence to the fall risk reduction algorithm	‘Green Pass System’ to prevent infant abduction, ensuring that the infant security sensor is removed only at the final step of discharge	Not all outcomes are reported; limited generalizability
[28]	Saudi Arabia	Cross-sectional (nursing perception)	RFID technology for infant tracking and prevention of mismatching, swapping, and abduction	OB/GYN department; 190 nurses	Effectiveness of RFID in infant tracking, identification accuracy, nursing staff acceptance, and perceived workflow improvements	65.8% of nurses perceived RFID effective for tracking; 62.1% agreed on identification accuracy; only ~50% accepted the system; 39% noted workflow improvements; inadequate training and computer skills affected acceptance	The strength of the RFID wristband fulfills the first goal of the Joint Commission’s National Patient Safety Goals, which is improving patient identification	Low tech skill, limited training, affects implementation
[29]	India	RFID system design and prototype test	RFID (tags + modules)	Neonatal/maternity wards	System accuracy in infant detection, response to RFID tampering/removal, alarm effectiveness during unauthorized activities	Achieved 100% accuracy in detecting infants and triggering alarms in simulated scenarios; demonstrated effective infant security monitoring	Not stated	Needs field validation; security, scalability concerns
[30]	Sri Lanka	Qualitative policy/legal review	Electronic security (recommended)	Hospitals; abducted infants for adoption	Impact of laws and guidelines on infant abduction and illegal adoption, analysis of reported cases, and consequences for victims/families	Despite existing laws, infant abduction and illegal adoption remain significant issues due to legal loopholes, inadequate hospital regulations, poor knowledge among relevant authorities, unreported cases, high legal costs, and a lack of legal aid for victims	Penal Code (Amendment) and particularly from the National Centre for Missing & Exploited Children (NCMEC)	Unreported cases; lack of legal aid; enforcement gaps
[31]	France	Engineering simulation and modeling	Active RFID (UHF + LF)	Maternity wards; Infants wearing anti-kidnapping RFID tags, including during water immersion (e.g., baths)	Reliability of RFID tags when immersed in water, radio coverage prediction, electric field radiation, propagation loss, probability of RF link establishment	Water immersion minimally affects RFID tag reliability; the RF-LF detection system remains reliable; immersion causes random effects on the radiated field, but does not fully attenuate signals; RSSI-based triangulation is affected by random variations, leading to localization imprecision and false alarms	Not stated	Difficult to precisely model the electric field due to unknown variables (bathtub size, tag position/orientation); study limited to 2D floor plane; LF signal reliability is less detailed; findings depend on the detection strategy used
[32]	Taiwan	System deployment and evaluation	Active RFID tags with RSSI for real-time infant tracking and identification	Hospital; newborns	Ratio of 24 h rooming-in care, system effectiveness in preventing ID errors, compliance with Baby-Friendly Hospital Initiative (BFHI) standards	RFID system improved infant safety, prevented identity errors and theft, increased 24 h rooming-in rates, and supported hospital BFHI certification	The infant rooming-in tracking system addresses regulatory and accreditation requirements primarily through adherence to the Baby-Friendly Hospital Initiative (BFHI) standards, including specific 24 h rooming-in rate criteria, and supports the broader goal of patient safety as emphasized by organizations like JCAHO	Not explicitly discussed; possible limitations include RFID accuracy, cost, and technical failures
[33]	India	Biometric system development	Online biometric system using digital footprint imaging and advanced image processing (gray conversion, particle filtering, texture extraction)	Hospital; newborns	Recognition accuracy, False Reject Rate (FRR), False Acceptance Rate (FAR), ROC curves	The online footprint system showed much higher accuracy than traditional offline methods (1–20% accuracy). Robust against background variation and newborn movements, offering improved newborn security potential.	Not stated	Challenges in obtaining high-quality images due to newborn movement and background interference. Limited sample size and specific hospital environment limit generalizability
[34]	India	Experimental development of face recognition algorithms tailored for newborns	Face recognition using SURF and LBP algorithms	Newborn infants (database of 280 faces)	Face recognition accuracy: impact of facial expressions on recognition	Achieved 87.04% accuracy; disproved the assumption that newborn faces are indistinguishable	Not stated	Difficulties capturing neutral facial expressions; newborn non-cooperation; limited and less diverse image database
[35]	Not specified	Multidisciplinary study developing intelligent infant face recognition using neural networks	Classifiers: Neural networks, Support Vector Machines (SVMs), Norm classifiers; dimensionality reduction via PCA and FLDA	Infants (150 individuals with pose, illumination, and expression variations)	Recognition performance; classifier comparison	SVM outperformed neural networks by >10%; demonstrated the feasibility of infant face recognition	Not stated	Challenges due to infant non-cooperation, variable poses and expressions; time-consuming image acquisition; limited infant databases; privacy and parental consent issues
[36]	Not specified	Descriptive study analyzing fetal abduction cases using secondary data from the media	Secondary interpretive data analysis	Fetal abductors (convenience sample)	Crime characteristics and motivations	Fetal abductors are often older than victims; typically married or in relationships; motivations linked to the need for attention and relationships	Not stated	Reliance on media reports with possible bias; lack of official data; unknown true population; no direct perpetrator interviews; exclusion of male conspirators
[37]	Japan	Observational study on maternal visual monitoring behaviors in free-ranging Japanese macaques	Behavioral observation (focal sampling)	171–194 Japanese macaques, including mothers and infants	Infant monitoring rate by maternal behavior, infant age, and mother-infant distance	Monitoring declines as the infant ages; mothers monitor more when infants are further away or handled by others; trade-offs exist between monitoring and other activities	Not stated	Potential collinearity between variables; exclusion of short-duration data; limited sample size
[38]	Not specified	Evaluation of RFID electromagnetic field exposure in mother-newborn identity reconfirmation	Passive RFID system compliance with ICNIRP EMF exposure guidelines	Scaled newborn and mother anatomical models	Maximum permitted magnetic field thresholds and time of use compliance	Newborns exposed to higher EMF levels; compliance depends on exposure time and device specs; training reduces overexposure risk	Not stated	Use of scaled models may not perfectly reflect real newborn anatomy; reader position variability affects exposure; limited real-world validation
[39]	Not specified	Description of a novel Infant Identifier biometric tool linking infant saliva and maternal fingerprint	Biometric Infant Identifier tool capturing infant saliva (DNA and scent) plus the mother’s fingerprint on a thermoplastic wafer	Hospitals and healthcare facilities, Infants and mothers	Effectiveness in linking infant and mother; enhanced infant security	Enables quick, non-invasive identification; supports scent dog tracking for missing infants; promotes infant security in healthcare	American Academy of Pediatric Dentistry (AAPD), Federal Bureau of Investigation (FBI), and JCI emphasize the necessity of robust infant identification programs	No large-scale validation reported; technology acceptance and privacy concerns not addressed
[4]	United States	Comparative analysis (1983–1992 vs. 1993–2006); 72 court cases + NCMEC/FBI data	Focus on abduction cases and prevention guidelines from NCMEC	Hospitals, birth centers, pediatric clinics 247 abductors; 96% female; 41% Black, 39% White, 20% Hispanic; ages 14–53	Infant recovery time, injury rates, and weapon use	Increase in Hispanic abducted infants and weapon use over time; rise in parental injuries; improved infant recovery rates within 24 h in healthcare settings; NCMEC guidelines and training support hospital safety improvements	National Center for Missing and Exploited Children (NCMEC), Association of Women’s Health, Obstetric, and Neonatal Nurses, and National Association of Neonatal Nurses involve staff training, implementation of security measures, and creation of prevention and response policies in facilities	Secondary data may introduce bias or underreporting; limited qualitative insights into abductor motivations; missing data in some demographic fields; findings may not generalize beyond the U.S. context
[40]	Not specified	Technology assessment	RFID (Radio-Frequency ID); encryption, LAN isolation, tamper-proof strap	Hospitals, birthing centers	Identification of vulnerabilities in RFID systems; development of technical security recommendations (e.g., password protection, LAN isolation, cryptographic upgrades); emphasis on staff training needs; call for improved surveillance and alarm protocols	RFID systems have critical vulnerabilities; need for improved encryption, network isolation, stronger passwords, tamper-proof tags, camera surveillance, and staff training; recommended protocols can mitigate risks	Not stated	Limitations in RFID technology create security gaps; reliance on technology without adequate human training may increase false alarms and security breaches; current systems do not fully address vulnerabilities
[41]	Not specified	Safety protocol overview	Infant security tag/abduction alarm system that triggers alarms, locks doors, and freezes elevators when infant nears exits or elevators; foot printing, color photos, and physical exam documentation within 2 h of birth; staff ID badges and double identification for infant-contact staff	Hospitals and healthcare settings with newborn units	Sentinel events reported to The Joint Commission (1983–2004); percentage and number of infant abductions; effectiveness of security protocols	Infant abductions are rare (0.5% of sentinel events); 116 infants were abducted over 21 years. Security alarms and protocols help prevent abduction attempts; parental and staff awareness are crucial for safety	JCI regulatory requirements involved wrist and ankle bands, Footprint, security tag or abduction alarm system, Control access to the maternity unit, and require staff ID	Low incidence may reduce perceived threat
[42]	United States	Quasi-experimental; archived FBI/NCMEC case review	Not specifically addressed (focus on security standards by JCAHO, including personnel orientation, patient/staff ID, and access control)	Hospitals, homes, other offenders (violent/deceptive); diverse race; 1985–2001	Abduction method (force, deception, theft); race differences	Force is more likely among offenders with a violent history; race disparities	Joint Commission for the Accreditation of Healthcare Organizations (JCAHO) Involved Personnel Orientation and Continuing Education, Identification of Patients and Staff, and Access Control	Small sample size; data biases; limited mental health records
[43]	United States	Policy review	Legislative focus on RFID/cybersecurity	healthcare	Legislative review of U.S. congressional bills related to cybersecurity and infant abduction; identifies emerging interest in regulating technological safeguards in healthcare, though without empirical data or implementation outcomes	The study highlights growing legislative attention in the U.S. to cybersecurity and infant abduction prevention through congressional bills. It reflects federal concern regarding technology vulnerabilities (e.g., RFID systems) in healthcare and calls for stronger regulatory frameworks and national standards to protect infants	The Health and Human Services Department (HHS) required hospitals to have security procedures designed to reduce the likelihood of infant abduction and infant switching	The paper does not include empirical data, implementation outcomes, or stakeholder input. It lacks evaluation of policy effectiveness, healthcare setting applicability, or direct links between legislation and clinical practice
[44]	United States	Descriptive/Review	Infant security systems: CCTV with recording, access control, infant bracelet/umbilical cord alarms; combined with traditional ID methods	Hospitals, maternal-newborn units, Female abductors (20–39 y/o), often overweight, manipulative, local, sometimes recently miscarried	Incident reports of abductions and near-misses; location of abduction; security protocol adherence	Infant abductions are rare but preventable; 80% occur in postpartum rooms or nurseries; no violent abductions since 1996; vigilance and technology integration are critical; multidisciplinary task forces improve security planning	International Association for Healthcare Security and Safety (IAHSS) and the National Center for Missing and Exploited Children (NCMEC) involved the requirement of ‘hardening the target’ in addition to the Health Care Financing Administration of financial penalties	No quantitative effectiveness data; focused on policy and security recommendations; limited generalizability outside the U.S. context
[45]	United States	Case report/Policy review	Electronic security systems (e.g., infant ID protocols, access control, birth data management, mock drills) are mandated by JCAHO	Hospital newborn/birthing units	Incident response success; implementation of JCAHO-mandated root cause analysis and action plans; emergency response plan effectiveness	Emergency response plan led to successful infant recovery; JCAHO standards improved hospital safety; no abductions by non-family reported in 1999; regular drills and education enhanced staff preparedness	Emergency management plans for newborn abduction must be in place in every institution that seeks accreditation from the Joint Commission on Accreditation of Healthcare Organizations, which requires multidisciplinary planning of critical incident response procedures, staff education, mock newborn abductions, and evaluation of response during the mock incidents	No empirical data or quantified outcomes; country not specified; limited generalizability; emotional impact noted but not systematically evaluated
[46]	United States	Case study/Incident analysis	Infant sensor bracelet and security door system (both failed in incident)	Hospitals, Hospitalized infants, hospital staff	Infant sensor bracelet and security door (both failed)	Security system failures led to infant abduction; highlights technology vulnerability; accreditation by Joint Commission influences hospital safety policies; security measures essential but costly and can limit patient access	JCAHO, Health Care Financing Administration (HCFA), and The Hospital Licensing Act specifically requires that hospitals create procedures addressing infant abductions, involving Architectural plans for controlling access to infant care areas, video surveillance, and staff/visitor identification protocols	No quantitative data; case-specific; limited generalizability; no detailed evaluation of accreditation effectiveness; emphasis on policy rather than empirical outcomes

Mandate (explicit citation of a standard, regulatory requirement, accreditation element, or compliance audit requirement).

**Table 3:** Frequency of technologies used across included studies.

Technology Category	Frequency	References	Percentage (%)
RFID systems/RFID tags/RFID technology	11	[3,24,26,28,29,31,32,38,40,41,43]	40.7%
Biometric systems (footprint, face recognition, saliva, fingerprint)	6	[27,32,33,34,39,41]	22.2%
Computer vision/machine learning/deep learning	3	[22,33,34]	11.1%
Surveillance cameras/CCTV	2	[3,44]	7.4%
Simulation technology/drills	3	[3,23,45]	11.1%
Security protocols/SOP development	2	[40,41]	7.4%
Sensor bracelets/infant sensor systems	3	[26,41,46]	11.1%
Physical security measures (locks, doors, alarms)	3	[3,41,44]	11.1%
Risk assessment/FMEA	1	[26]	3.7%
Legislation/Policy review	2	[30,41]	7.4%
Others (e.g., engineering modeling, observation)	3	[31,37,38]	11.1%

## Data Availability

Not applicable.

## Supplementary Materials

The following supporting information can be downloaded at: https://www.mdpi.com/article/10.3390/healthcare14030377/s1, File S1: Check-list; File S2: Search Strategy; File S3: Critical Appraisal. Reference [47] has been mentioned in the text.

## Registration Information

This systematic review is registered in the International Prospective Register of systematic reviews (PROSPERO) at the National Institute for Health and Care Research https://www.crd.york.ac.uk/PROSPERO/view/CRD420251078622 (accessed on 30 November 2025).
