# Supplementary material for: Ensuring Safe Newborn Delivery Through Standards: A Scoping Review of Technologies Aligned with Healthcare Accreditation and Regulatory Frameworks
Source: Healthcare (Basel). 2026 Feb 2;14(3):377. doi: 10.3390/healthcare14030377 (PMC12897065; doi:10.3390/healthcare14030377)
Supplement: Supplementary file 1 [file healthcare-14-00377-s001.zip › Supplementary file 3 - Critical appraisal.pdf]

Supplementary file 3 - Risk of Bias Assessment

AACODS Checklist

Reference	Q1	Q2	Q3	Q4	Q5	Q6
(Shilaskar et al., 2024)	Y	Y	N	Y	Y	Y
(Lehmann, 2024)	Y	Y	Y	Y	Y	Y
(Pandya et al., 2023)	Y	Y	Y	Y	Y	Y
(Kiruthiga et al., 2023)	N	Y	N	N	Y	Y
(Batool et al., 2023)	Y	Y	N	Y	Y	Y
(Bittle & Scalise, 2017)	Y	Y	N	Y	Y	Y
(Al Osaimi et al., 2017)	Y	Y	Y	Y	Y	Y
(Samayawardena, 2015)	Y	Y	Y	Y	Y	Y
(Crémoux et al., 2014)	Y	Y	Y	Y	N	Y
(Hung et al., 2013)	Y	Y	Y	Y	N	Y
(Balameenakshi et al., 2013)	Y	Y	N	Y	N	Y
(Tiwari & Singh, 2012)	Y	Y	Y	Y	N	Y
(Tiwari et al., 2012)	Y	Y	Y	Y	N	Y
(Arquette, 2012)	Y	Y	Y	Y	N	Y
(Tesini & RCSE, 2009)	Y	Y	Y	Y	N	Y
(Burgess et al., 2008)	Y	Y	Y	Y	N	Y
(Saad & Ahamed, 2007)	Y	Y	Y	Y	N	Y
(Miller, 2007)	Y	Y	Y	Y	N	Y
(Strohman, 2005)	Y	Y	Y	Y	N	Y
(Anderson, 2005)	N	N	Y	N	N	Y
(Cesario, 2003)	Y	Y	Y	Y	N	Y
(Shogan, 2002)	Y	Y	Y	Y	N	Y
(Goodwin, 2001)	Y	Y	Y	Y	N	Y

SQUIRE 2.0 Checklist

Reference	Q1	Q2	Q3	Q4	Q5	Q6	Q7	Q8	Q9	Q10	Q11	Q12	Q13	Q14	Q15	Q16	Q17	Q18
(Webster et al., 2021)	Y	Y	Y	Y	Y	Y	Y	N	Y	N	Y	Y	Y	Y	Y	Y	Y	Y

TIDieR Checklist

Reference	Q1	Q2	Q3	Q4	Q5	Q6	Q7	Q8	Q9	Q10	Q11	Q12
(Yadav et al., 2016)	Y	Y	Y	Y	N	Y	Y	Y	Y	N	N	N
(Fiocchi et al., 2011)	Y	Y	Y	Y	N	N	Y	Y	Y	N	N	Y

JBI cross-sectional analytical studies

Reference	Q1	Q2	Q3	Q4	Q5	Q6	Q7	Q8
(Onishi, 2011)	Y	Y	Y	Y	Y	Y	Y	Y

Y=YES, N=No
