# Supplementary material for: Ensuring Safe Newborn Delivery Through Standards: A Scoping Review of Technologies Aligned with Healthcare Accreditation and Regulatory Frameworks
Source: Healthcare (Basel). 2026 Feb 2;14(3):377. doi: 10.3390/healthcare14030377 (PMC12897065; doi:10.3390/healthcare14030377)
Supplement: Supplementary file 1 [file healthcare-14-00377-s001.zip › Supplementary_file_2-Search_Strategy.pdf]

Supplementary file 2 - Search Strategy

PubMed 2000 - 2024

((newborn*[Title/Abstract]) OR (neonat*[Title/Abstract]) OR (maternity unit[Title/Abstract]) OR ("Infant, Newborn"[Mesh]) OR (obstetric*[Title/Abstract]) OR (delivery room[Title/Abstract])) AND ((RFID[Title/Abstract]) OR ("Radio Frequency Identification Device"[Mesh]) OR (barcode*[Title/Abstract]) OR (bar code[Title/Abstract]) OR (biometric*[Title/Abstract]) OR (electronic bracelet[Title/Abstract]) OR (smart ID band[Title/Abstract]) OR (infant security system[Title/Abstract]) OR (infant tagging[Title/Abstract]) OR (mother-infant matching[Title/Abstract]) OR (electronic infant verification[Title/Abstract]) OR (real-time location system[Title/Abstract]) OR (mobile newborn tracking[Title/Abstract])) AND ((safe delivery[Title/Abstract]) OR ("Patient Safety"[Mesh]) OR (mismatch*[Title/Abstract]) OR (identification error[Title/Abstract]) OR (abduction*[Title/Abstract]) OR (wrong baby[Title/Abstract]))

Web of Science 2000 - 2024

TS = ((newborn* OR neonat* OR "maternity unit" OR obstetric* OR "delivery room") AND (RFID OR "radio frequency identification" OR barcode* OR biometric* OR "electronic bracelet" OR "smart ID band" OR "infant security system" OR "mother-infant matching" OR RTLS OR "real-time location system" OR "mobile newborn tracking" OR "electronic infant verification") AND (accreditation OR regulation* OR standard* OR guideline* OR mandate*) AND ("safe delivery" OR "patient safety" OR mismatch* OR abduction* OR error* OR "wrong baby"))

Google Scholar 2000 - 2024

("newborn" OR "newborns" OR "neonate" OR "neonatal" OR "maternity unit" OR obstetric OR "delivery room") AND (RFID OR "radio frequency identification" OR barcode OR "bar code" OR biometric OR "electronic bracelet" OR "smart ID band" OR "infant security system" OR "infant tagging" OR "mother-infant matching" OR "electronic infant verification" OR RTLS OR "real-time location system" OR "mobile newborn tracking") AND (accreditation OR standards OR regulations OR policy OR mandate OR guideline) AND ("safe delivery" OR "patient safety" OR mismatch OR "identification error" OR abduction OR "wrong baby")
